# The Medical Regulator Again

**Published:** 1857-11

**Authors:** 


### The Medical Regulator Again.

Dr. Bowling, of the Nashville Journal, has thought proper, a second time, in the exercise of his powers as regulator of American Medical affairs, and especially of the press, to introduce the name of one of the Editors of this Journal in an offensive way, into the columns of *his* Journal, as we believe he calls it. Now, though we have but little to say in reply to the foul emanations which are constantly issuing from that source, that little, must be very decided.

In reference to the last *issue*, we pronounce it *untrue* that we have employed *any* part of our time in abuse of the American Medical Association; so far from it; we are to-day a better friend of that institution than the individual referred to. It is true, that we have, in the exercise of the ordinary privileges of a freeman though fit to express our opposition to the perversion of the Association from its proper and legitimate objects—the advancement of medical science and the promotion of harmony in, and elevation of the profession—to the pursuit of visionary or selfish schemes.

We have been presumptuous enough to attempt to discuss the powers of the American Medical Association in connection with the rights of individual members of the profession and those of State organizations, and instead of an attempt to refute our positions in a courteous and gentlemanly way, we have been met by an insufferable arrogance and insolence to which we do not feel disposed to submit.

Now, in conclusion, we would remark that we do not know that we have any special interest in maintaining friendly relations with "A Western Editor," but we do feel a special interest in saying to him that we shall continue to despise and denounce *hypocrisy*, even though it present itself under the specious garb of *diplomacy*.

### *The Presidency of the American Medical Association.*

It is evident that the time has arrived when some change in the manner of selecting the presiding officer of our national congress must be made. We have felt for years that some one should speak out upon the subject. We would have spoken a year ago, had not the fact that the meeting of the association, for 1856, was to be held at Detroit, and that the candidate then most prominent and since successful, has been in a position of personal antagonism with an interest which we represent, prevented us. Any attack made by us, at that time, would have been construed as personal, and as having its origin in local prejudices or jealousies; we therefore held our peace, and consented to forego our protest against an evil which must eventually impair the respectability of the association, and turn from it the regard of the best men in the profession.

The next meeting is to be held at Nashville. We have none but friends in that locality, and we believe that they, too, will acquiesce in our plans for reform, or help to suggest another looking to the same end.

The evil to which we allude is self-evident. Custom, not law, has made it the usual course to select the president of the association from the city in which the session is held. Thus the two meetings held at Philadelphia have given us Profs. Chapman and Wood, as presidents; that in St. Louis, Prof. Poe; that in Richmond, Dr. R. B. Wellford, and that in Detroit, Dr. Z. Pitcher. In only one instance has the rule failed to hold good. At the meeting in New York, in 1853, no one worthy of the office was found in that great city, and Dr. Jonathan Knight, of New Haven, was chosen. We hoped then that the precedent thus established in a large city, might be permitted to hold good in the small towns; but it is not so to be. It is evident, that until it is put out of the power of the nominating committee to virtually elect the president, we shall forever be subjected to the rule of that happy individual who happens to be the Nestor of the place of meeting. Grey hairs are honored at the expense of working men. Worse than this. To follow back the chain of promotion, we shall find that the association take the man the nominating committee chose to give them, and the committee take the man they are instructed to by the wire-pullers of the particular locality. In a small town, with some forty to fifty physicians, this matter can usually be cozily arranged among themselves, with more regard to their own interests than to the dignity or the association.

We regard the presidency of the American Medical Association as an honor too high to be lightly bestowed upon any man. The consideration of locality is the last that should be thought of. It is nonsense to make it a mere compliment to a town, in return for a good feed and abundant champagne. The occupant of that high station should be chosen from the profession at large. He who has labored; he who, through many years, has toiled to advance his paofession; he who has built up for himself a name as a discoverer or medical philosopher; and who, perhaps, has found no other reward than a good name for all his efforts, such an one is our candidate.—*Buffalo Med. Journal.*—*Nashville Journal.*

We endorse without hesitation the views of the Buffalo Journal. * * We believe that the nomination of this officer shouldbe taken from the Nominating Committee, and that he should be elected by the Association by ballot. In no other way can the presiding officer be regarded as the representative of the Association. In this way the promotion of a member to this exalted office would be to confer upon him the highest honor in the power of his professional countrymen. This plan would secure the attendance of the most distinguished of our brethren, for the selection would necessarily be made from those present. But when it is nnderstood beforehand that this office is rather the result of the accidental conjunction of the place of meeting and place of residence, the position cannot be ragarded of a distinguished honor. Every physician who loves his profession must be ambitious of the good opinion of the more high-minded and honorable of his brethren, and cannot be indifferent to such honors as they can through their public meetings and associations confer upon him, and to this extent every one may be regarded as feeling more or less personally interested in the presidency of our Association, the very highest position which our suffrage can secure to the worthy among us. The framers of our constitution could not have foreseen the influence of local circumstances in determining the presidency of the Association, but as experience clearly shows that such is the fact, it is apparent that it is now the first great dutyof this body to so amend the constitution as to secure a more just and equitable method of appointing its president. But as the present provisions cannot be repealed at the next meeting, a constitutional provision requiring the resolutions to change the instrument shall lay over to the next meeting, our brethren abroad may very naturally expect in advance some expression of the feelings of the profession in the State upon that point. Those with whom we have discussed this subject here agree that the *habit* of the Association in selecting its presiding officer from the city in which the meeting occurs, is a bad one, and cannot fail to destroy the dignity and honor of the position, and consequently impair the honor, dignity and usefulness of the Association, and that the sooner a bad habit is corrected the better, and do therefore most earnestly hope that the reformation may begin at Nashville at the next meeting of the Association —*Nashville Journal.*

We present above the position of the Nashville Journal in reference to the Presidency of the American Medical Association in October preceding the meeting in Nashville ; notwithstanding which, one of the Editors became the President of the body, with the concurrence, (as we have a right to presume from his own statement in the September number of this year,) of the other, who had taken such decided ground against the selection of the President of the Association from the place at which the meeting was held, for which course, as he says, "Dr. Eve also declared."

Now the excuse for this, *apparently* double faced operation is, (according to Dr. Bowlings defence,) "a double game of Dr. Gross," of Philadelphia, who had expressed himself in favor of selecting the Presiding Officer of the Association from some other locality than that occupied by the body, but who was afterwards found "advising and endeavoring with a fair prospect of success to secure the election of a Tennesseean to that office."

A change in the views of Dr. Gross, made the selection of one of the Editors of the Nashville Journal, an indispensable necessity, they being the only men in Tennessee who could be even *thought of* as deserving that position. Comment upon such logic is useless.

Now, we mean for it to be remembered, that in this matter we have said nothing intended to be personally offensive towards Dr. Eve or in any way disrespectful of him—we have not said that *he* had any agency in bringing himself before the nominating committee as a candidate for the office of President of the Association, but we do say that the Editor of the Nashville Journal has placed not only himself but his assistant in a very unenviable attitude in reference to this subject, to which however we could have had no disposition to allude, but for the dictatorial and offensive course of this inflated boaster.

